# Seborrheic Keratosis Through the Lens of Histopathology: A Case Report

**DOI:** 10.7759/cureus.95780

**Published:** 2025-10-30

**Authors:** Mehebuba Sultana, Arindam Mandal, Arunit Chatterjee, Sanjeet K Das, Sangeeta Sinha, Rudra Prasad Chatterjee

**Affiliations:** 1 Oral and Maxillofacial Pathology, Guru Nanak Institute of Dental Sciences and Research, Kolkata, IND; 2 Maxillofacial Surgery, Desun Hospital, Kolkata, IND

**Keywords:** benign, keratin plugging, keratosis, pigmented lesion, pseudocyst, seborrheic

## Abstract

Seborrheic keratosis (SK) is the most common benign pigmented epidermal lesion, typically occurring in elderly individuals and mostly confined to sun-exposed areas. Though SK is harmless, it can mimic both benign and malignant cutaneous lesions, necessitating histopathological evaluation for accurate diagnosis. The lesion commonly presents as a pigmented, verrucous growth with clinical suspicion requiring further investigation. We report a case of SK involving the right cheek region in an elderly female. The lesion was managed successfully, and the patient had an uneventful recovery. This case highlights the importance of recognizing the clinical and histopathological diversity of SK to ensure precise diagnosis and appropriate treatment.

## Introduction

Seborrheic keratosis (SK) is the most common benign pigmented epidermal lesion seen in the elderly population [1,2]. According to a study by the US National Health and Nutrition Examination Survey (NHANES), about 23 million persons in the United States are affected with SK. It was first demonstrated in the year 1869, as a well-circumscribed, black, round, elevated, *stuck-on* skin lesion that increases with age [1]. SK is also known as senile wart, melanoacanthoma, basal cell papilloma, senile keratosis, seborrheic wart, verrucosa seborrheica, or benign acanthokeratoma [3]. Most of SK appears on sun-exposed skin, i.e., face, trunk, chest, and back; that’s why the manifestation of this lesion on the mucous membrane is restricted [3,4]. Clinically, they appear as papular or verrucous corn-like pigmented growth with overlying greasy scale [3]. Histopathologically, these lesions appear as hyperproliferation of immature keratinocytes from the basal layer, mostly containing melanocytic pigmentation [2,4]. SK reveals morphological similarities with other benign and malignant lesions; therefore, histopathological investigations are highly recommended for accurate diagnosis [4]. Here, a case report of SK over the right cheek region in an elderly female has been discussed in this article with detailed clinical, histopathological features, along with treatment procedure, with special emphasis on its histopathological diversity.

## Case presentation

**Figure 1:**
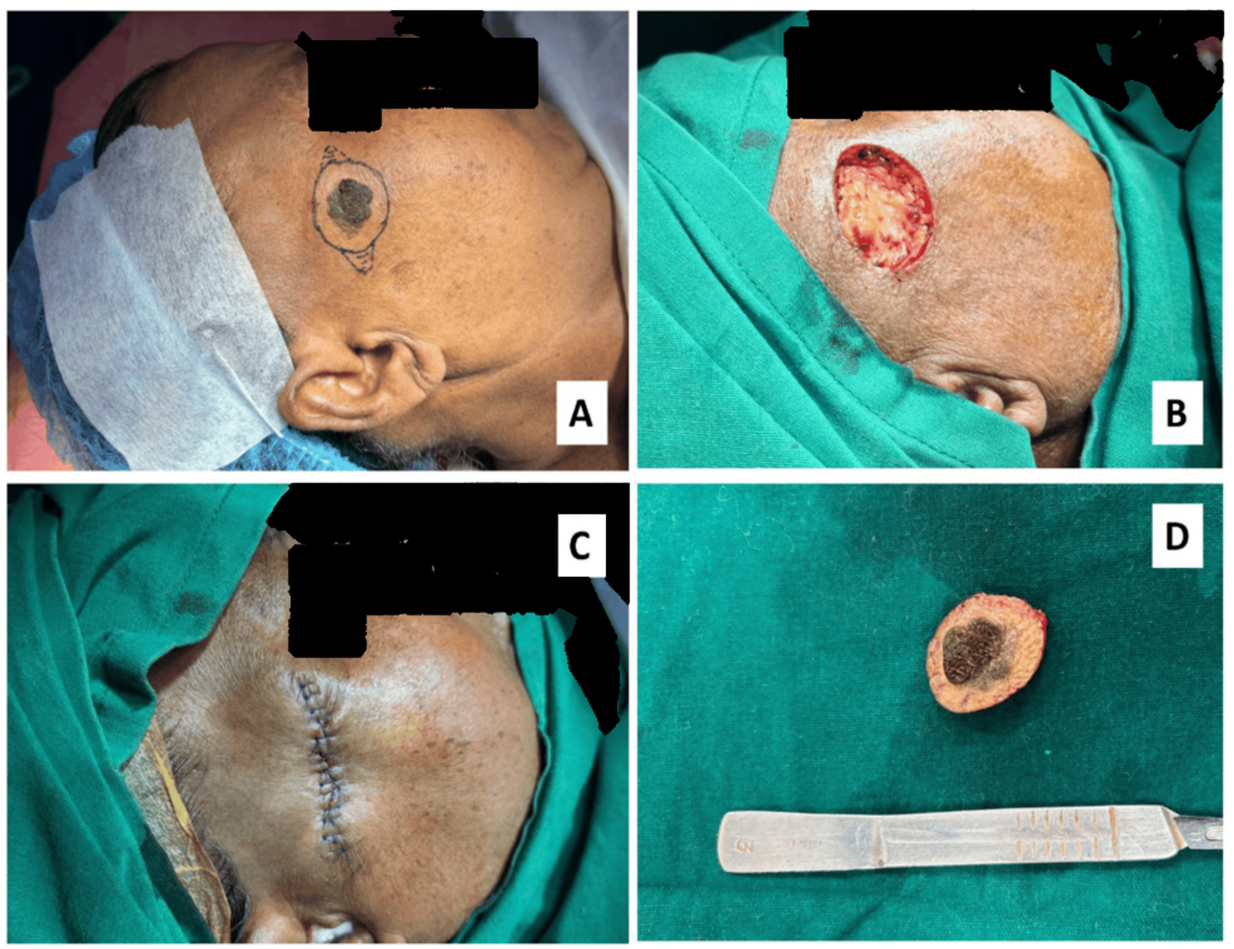
Clinical images showing (A) a solitary, well-demarcated, brownish nodular growth lateral to the right canthus, marked with a skin marker; (B) an incision made with a 1 cm margin of healthy skin; (C) closure of the defect with sutures; and (D) the gross specimen.

A 56-year-old Indian female reported with a painless, brownish, pigmented growth involving the right temple region for the last three years, which was progressively increasing in size with symptoms of discomfort for the last four months. The lesion was situated 2 cm lateral to the right canthus and 1 cm below the hairline, measuring around 1 cm x 0.75 cm. On examination, the lesion was a solitary, well-demarcated, brownish nodulopapular growth without tenderness or discharge. The overlying surface was fissured, rough, leathery, and firm in consistency (Figure 1A). A provisional diagnosis of epidermal nevus was made, with differential diagnoses including actinic keratosis, verruca vulgaris, seborrheic keratosis, and basal cell carcinoma.

Informed consent was taken, and an excisional biopsy was performed under local anesthesia to confirm the diagnosis. Following sterile preparation of the surgical site, the lesion was marked with a skin marker (Figure 1A). The incision line was planned along the facial relaxed skin tension lines (RSTLs) to minimize scarring. Local anesthesia and hemostasis were achieved by infiltrating the area with 2% lidocaine containing 1:100,000 epinephrine. A #15 scalpel was used to make the skin incision, which was then deepened with electrocautery in coagulation mode, maintaining a 1-cm margin of healthy skin around the lesion to prevent recurrence (Figure 1B). The skin edges were undermined to reduce tension on the closure. The defect was closed in two layers using 4-0 Vicryl for the deep layer and 6-0 Prolene for the skin (Figure 1C).

**Figure 2:**
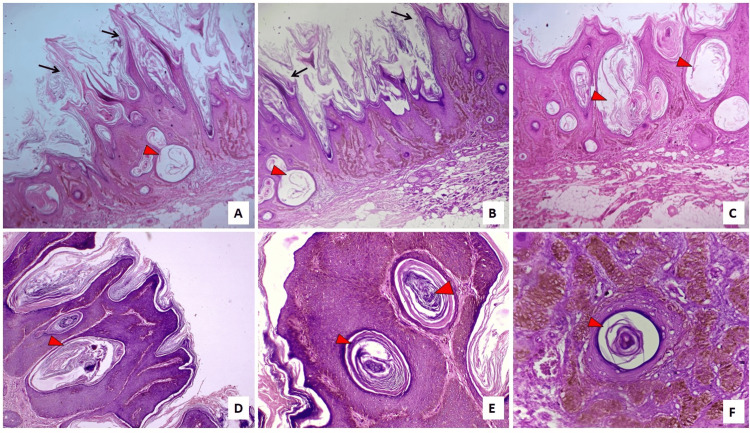
H&E-stained photomicrographs. (A-D) Low-power view (4×) showing a *church spire* appearance (black arrow) with orthokeratotic plugging and multiple intraepithelial keratin-filled pseudocysts (red arrowhead). (E) Medium-power view (10×) and (F) high-power view (40×) showing intraepithelial pseudokeratocysts (red arrowhead) and basal cell proliferation with melanotic changes.

Then the excised tissue was sent for histopathological examination (Figure 1D). The H&E-stained sections revealed a hyperplastic epidermis with papillary projections resembling a *church spire* appearance and marked orthokeratotic plugging (Figures 2A-2B). The presence of multiple intraepithelial keratin-filled pseudo-cysts was noted (Figure 2). Proliferation of atypical basal cells with melanotic changes (Figure 2F), along with juxtaepithelial focal presence of melanophages, was also noted. The superficial dermis revealed chronic non-specific inflammatory cell infiltration. After a thorough clinicopathological evaluation, the final diagnosis of the hyperkeratotic type of seborrheic keratosis was concluded.

## Discussion

SK is a common benign epidermal tumor. Most commonly arises in individuals older than 50 years without any sex predilection. In our case, the patient's age was in accordance with other reported cases. Clinically, it presents as a sharply demarcated, slightly raised, brownish patch or plaque, mainly in sun-exposed areas except the palms and soles, i.e., trunk, particularly the interscapular area, sides of the neck, face, and arms. The present lesion was noted over the temporal region of the face, likewise previous cases. Moreover, this warty lesion shows higher occurrence in skin phototypes I and II [1,2,5]. Morphologically, some variants of SK have been described, including the common flat type (solar lentigo), skin tag-like, stucco keratosis, dermatosis papulosa nigra, inverted follicular keratosis, large cell acanthoma, lichenoid keratosis, and melanoacanthoma. Previous studies have rarely reported giant SKs involving the genital, perianal, or facial regions [4,6].

SKs are usually easily identified on clinical examination; however, some lesions may mimic conditions such as common warts, lentigines, actinic keratosis, verruca vulgaris, Bowen’s disease, keratoacanthoma, and more aggressive entities like basal cell carcinoma, squamous cell carcinoma, and cutaneous melanoma [1,2,5].

Despite its high occurrence, the proper etiopathogenesis of this lesion is still not understood. Hence, heredity, sun exposure, chronic irritation, and human papillomavirus (HPV) have been suggested as risk factors. According to recent genetic studies, somatic mutations in the Fibroblast Growth Factor Receptor 3 (FGFR3) gene could be a considerable aspect for the development of SK [1,2]. The sudden appearance of multiple SKs may indicate the sign of Leser-Trélat (LT), which can be associated with an underlying malignancy [3].

**Figure 3:**
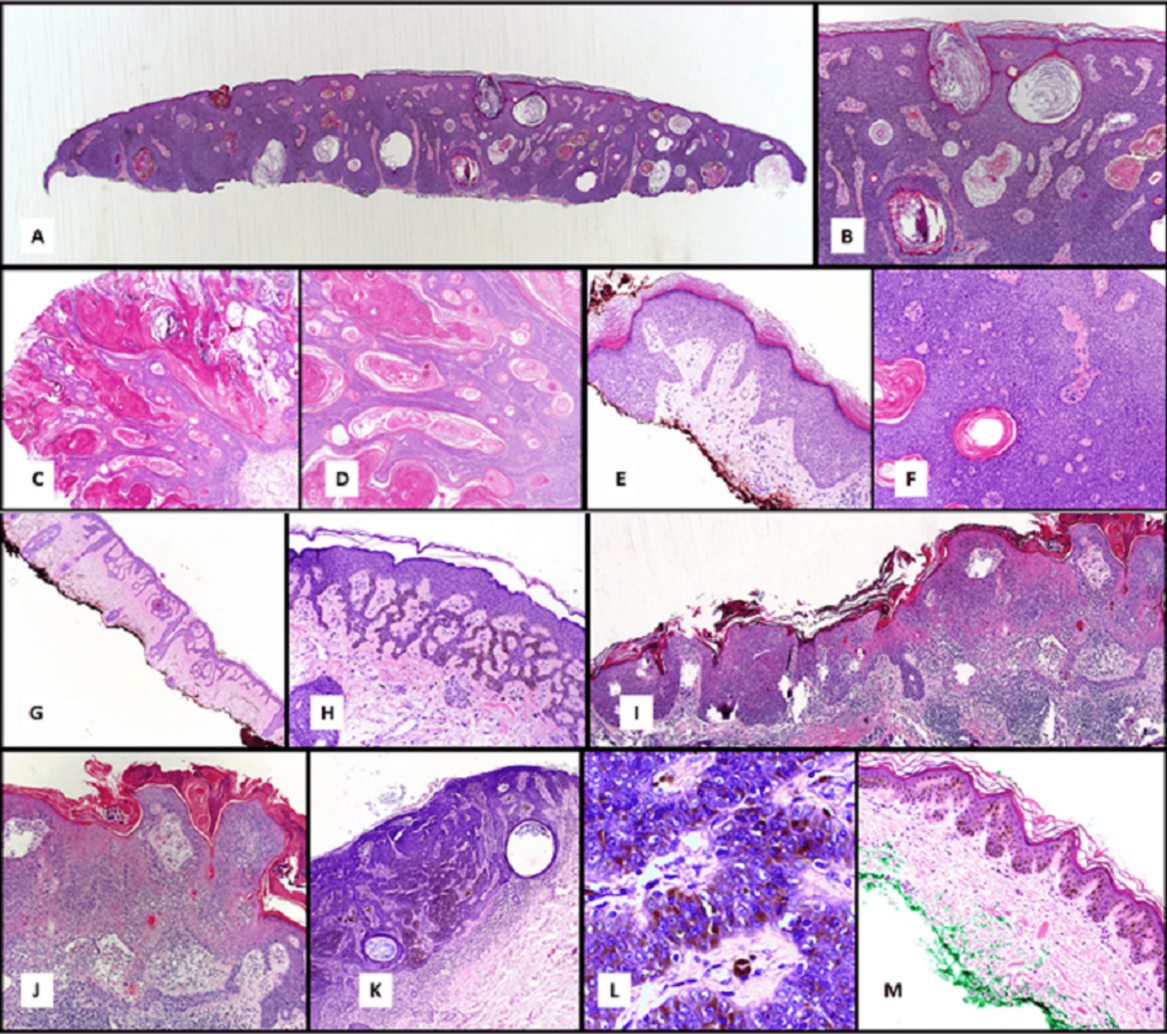
Photomicrographs showing various histopathological subtypes of seborrheic keratosis. (A) Low-power and (B) high-power views, acanthotic type; (C) low-power and (D) high-power views, hyperkeratotic type; (E) low-power and (F) high-power views, clonal type; (G) low-power and (H) high-power views, reticulated type; (I) low-power and (J) high-power views, irritated type; (K) low-power and (L) high-power views, pigmented type; and (M) low-power view, macular type. Image courtesy: PathologyOutlines.com; permission obtained to reproduce photomicrographs [7].

SK exhibits seven histopathological variants: acanthotic, hyperkeratotic, reticulated, clonal, irritated, pigmented (melanoacanthoma), and macular (Figure 3; Table 1). Acanthotic type is the highest occurring subtype, which demonstrates prominent acanthosis of basaloid cells with papillomatosis and hyperkeratosis. *Pseudo-horn cysts* and *true horn cysts* could also be seen in this type. Melanotic changes occur in about one-third of acanthotic cases. In sun-exposed areas, acanthotic-type SK can occasionally progress to an in situ carcinoma, known as Bowenoid transformation (Figure 3A, low power; Figure 3B, high power).

**Table 1:** Histopathological features in various subtypes of SK. SK, Seborrheic keratosis

Histopathological types	Features
1. Acanthotic	-Prominent acanthosis of basaloid cells with papillomatosis and hyperkeratosis -Presence of “pseudo-horn cysts” and “true horn cysts” - Melanotic changes can occur in about one-third of cases
2. Hyperkeratotic	- Elongated “church spire” like projections exhibiting a papillomatous or verrucous appearance -Prominent hyperorthokeratosis and mild acanthosis -Horn cysts and hyperpigmentation are unusual
3. Clonal (Nested)	- Proliferation of well-delineated intraepithelial nests of basaloid or pale cells(Borst-Jadassohn phenomenon) - cells may show prominent intercellular bridges
4. Reticulated (Adenoid)	- Proliferation of basaloid epidermal cells in a threadlike pattern, forming branching and interweaving within the dermis. - Hyperpigmentation is common
5. Irritated	- Presence of inflammatory cell infiltration in dermis -Presence of intraepithelial squamous swirls, composed of aggregated eosinophilic squamous cells - Basal cell layer degeneration, acantholysis, dyskeratosis, and spongiosis can also be noted
6. Macular	- Minimal acanthosis - Hyperpigmented basal layer - Horn and pseudocysts are usually not present
7. Pigmented (Melanoacanthoma)	- Acanthotic or reticular subtype with marked increase in melanocytes containing melanin.

The hyperkeratotic type shows elongated, *church-spire-like* projections with a papillomatous or verrucous appearance, characterized by prominent hyperorthokeratosis and mild acanthosis. Horn cysts and hyperpigmentation are unusual in this variant (Figure 3C, low power; Figure 3D, high power).

On histopathological evaluation, the present case was diagnosed as the hyperkeratotic subtype of SK.

In the clonal (nested) subtype, there is a proliferation of well-delineated intraepithelial nests of basaloid or pale cells (Borst-Jadassohn phenomenon), whereas in some cases, these nests consist of larger cells with prominent intercellular bridges (Figure 3E, low power; 3F, high power).

The reticulated (or adenoid) type exhibits proliferation of basaloid epidermal cells arranged in numerous thread-like, double rows within the epidermis and forms branching and interweaving within the dermis. Hyperpigmentation is common in this type of SK. In some cases, a relationship has been noted between solar lentigo and the reticulated subtype of SK (Figure 3G, low power; 3H, high power).

Irritated subtype is characterized by inflammatory cell infiltration, like a lichenoid reaction in the dermis and intraepithelial squamous swirls, composed of aggregated eosinophilic squamous cells. Most of these swirls consist of intraepidermal hair follicle-like structures. Squamous swirls can be differentiated from squamous horn pearls, found in squamous cell carcinoma, by their large number, small size, and well-circumscribed configuration. Some important histopathologic features, like basal cell layer degeneration, acantholysis, dyskeratosis, and spongiosis, can also be noted here (Figure 3I, low power; Figure 3J, high power).

Macular pattern exhibits minimal acanthosis and a hyperpigmented basal layer. Horn and pseudocysts are usually not found (Figure 3M, low power) [3,5,7-10].

Though pigmentation occurs in acanthotic and reticular subtypes, the hallmark of melanoacanthoma (pigmented subtype) is a marked increase in melanocytes containing melanin (Figure 3K, low power; Figure 3L, high power). This subtype is often clinically confused with some malignant pigmented lesions, i.e, malignant melanoma, pigmented basal cell carcinoma [2,3].

Usually, asymptomatic SK needs no treatment except for cosmetic correction; moreover, sometimes it may resolve on its own within a period of time [5]. Rarely, eruptive SK indicates an internal malignancy (usually gastric adenocarcinoma). In a few cases of the irritated subtype, it can progress to eczematous dermatitis around it. Also, in the eruptive and irritated type, keratosis may occur as a complication of medications like adalimumab, vemurafenib, dabrafenib, and some chemotherapy drugs [6]. However, the pigmented and clonal (cateriform) types of SK should be differentiated from pigmented malignant lesions such as basal cell carcinoma and malignant melanoma. In such cases, biopsy for histopathologic evaluation is the gold standard technique to rule out malignant lesions. Therefore, surgical excision followed by reconstruction is the most acceptable treatment modality to avoid diagnostic dilemma for pigmented skin lesions [1,2,5]. As our present case was pigmented, surgical excision was done, followed by histopathological evaluation.

Otherwise, some non-surgical treatment modalities are available, i.e., ammonium lactate and alpha hydroxyl acids, application of pure trichloroacetic acid, topical treatment with vitamin D and tazarotene cream (0.1%) to increase the apoptosis of keratinocytes, laser therapy, cryotherapy with liquid nitrogen [1,2,4,5,11].

Recurrence is very rare in the case of SK. According to, American Academy of Dermatology Association (AAD), SK lesion has a good prognosis with a low recurrence rate after surgical excision. Therefore, long-term follow-ups are necessary. Although we have noted no recurrence in our case after thorough follow-ups for one year. Though it rarely progresses towards malignancy, very few cases of malignant transformation are reported in the irritated subtype exclusively in the flexure region [1].

## Conclusions

SKs are common benign skin lesions that mostly appear in sun-exposed areas. It has restricted mucosal involvement. It frequently affects elderly individuals, mimicking a few malignant tumors, and is sometimes associated with an underlying malignancy. Biopsy and histopathological evaluation should be done to avoid diagnostic dilemmas, mostly in cases of pigmented lesions.

## References

[1] Salah B, Mahseeri M, Al-Ali Z, Gaith A, Aldwan T, Al-Rawashdeh B (2018). Giant perianal Seborrheic keratosis: a case report. Int J Surg Case Rep.

[2] Kumar SC, Sinha AK, Singh S (2018). Seborrahic keratitis: a benign pigmented skin lesion management. Int J Res Med Sci.

[3] Phulari RG, Buddhdev K, Rathore R, Patel S (2014). Seborrheic keratosis. J Oral Maxillofac Pathol.

[4] Sepehri N, babaniamansour S, Karkon-Shayan S, Majidi M, Atarodi A, Mohammadzadeh H, Talaiee M (2020). Giant seborrheic keratosis on the right flank part: a case report. J Skin Stem Cell.

[5] Li W-R, Lin L (2020). Seborrheic keratosis in a young woman: a mimicker of keratoacanthoma. Int J Dermatol Venereol.

[6] Oakley A. Seborrhoeic Keratosis. DermNet (2016). Seborrhoeic keratosis. https://dermnetnz.org/topics/seborrhoeic-keratosis.

[7] Underwood CIM, Boswell E (2025). Seborrheic Keratosis.

[8] D Sarma, S Repertinger (2008). Seborrheic keratosis: a pictorial review of the histopathologic variations. Internet J Dermatol.

[9] Jeong J, Kie JH (2021). Seborrheic keratosis in the auricle. BMJ Case Rep.

[10] Mullins CI, Boswell E (2025). Seborrheic keratosis. https://www.pathologyoutlines.com/topic/skintumornonmelanocyticsk.html.

[11] Zare P, Ramezani M (2023). Seborrheic keratosis in an adolescent: a rare presentation. Clin Case Rep.

